# Nurturing Bonds: Parental Attachment, Breastfeeding Attitudes and Parenting Styles in Greece

**DOI:** 10.1192/j.eurpsy.2025.2021

**Published:** 2025-08-26

**Authors:** A. Kondyli, I. Koutelekos, D. Briana, A. Zartaloudi

**Affiliations:** 1 University of West Attica; 2 MSc in General Pediatrics and Pediatric Subspecialties - Clinical Practice and Research; 3 University of Athens, Athens, Greece

## Abstract

**Introduction:**

The emotional bond a person forms with their parents during childhood is a crucial factor that influences the choices they make and the parenting style they adopt when they become parents themselves. Conversely, the parenting approach one chooses and the decisions made in raising their children will impact their future relationship with their child.

**Objectives:**

To explore (a) the type of emotional bond that the participants had developed with their own parents during their childhood, (b) their attitudes towards breastfeeding, and (c) their parenting style.

**Methods:**

A cross-sectional study was conducted using self-administered questionnaires completed by 862 parents—both mothers and fathers—who had received support from a private maternity and breastfeeding support center in Athens.

**Results:**

Participants who received higher levels of care from their parents during childhood were negatively associated with exclusive breastfeeding (p = 0.041), shorter durations of breastfeeding (p < 0.001), and a positive attitude toward breastfeeding beyond 12 months (p = 0.002). Mothers who received high care from their parents tended to adopt a more supportive parenting style (p < 0.001), in contrast to those who experienced high levels of control (p = 0.001). A supportive maternal style was positively associated with natural weaning (p = 0.018). In contrast, a more authoritarian maternal style was positively associated with non-exclusive breastfeeding (p = 0.012), abrupt weaning (p = 0.021), introducing solid foods as the first food (p = 0.001), parents and children not sharing the same room (p < 0.001), and the implementation of sleep training (p < 0.001). Maternal permissiveness was positively associated with not breastfeeding (p = 0.011), non-exclusive breastfeeding (p = 0.002), pacifier use (p < 0.001), introducing pureed foods as the first foods (p = 0.001), and the use of sleep training (p = 0.001). For fathers, a shorter duration of room-sharing with the child was significantly associated with a stricter parenting style (p = 0.023). The more children a mother had, the more likely she was to adopt an authoritarian or permissive parenting style (p < 0.001), and the same was true for fathers (p < 0.001). Additionally, older paternal age was positively associated with a more authoritarian parenting style (p = 0.001). An overall positive breastfeeding experience was associated with being less authoritarian, strict, or permissive (p = 0.003; p = 0.005; p < 0.001, respectively).

**Conclusions:**

Breastfeeding may act as a catalyst for parents to adopt a more supportive parenting style toward their children, regardless of the type of bond they developed with their own parents. This study could serve as a foundation for more extensive research on breastfeeding, early parental choices, attachment bonds, and parenting practices.

**Disclosure of Interest:**

None Declared

